# Kinetic Projectile Injuries Treated During Civil Protests in Los Angeles: A Case Series

**DOI:** 10.5811/cpcem.2021.7.52885

**Published:** 2021-10-19

**Authors:** Rachel C. Pearl, Sam Torbati, Joel M. Geiderman

**Affiliations:** Cedars-Sinai Medical Center, Department of Emergency Medicine, Los Angeles, California

**Keywords:** case series, kinetic projectile, crowd control, less lethal weapons, protests

## Abstract

**Introduction:**

During protests following the death of George Floyd, kinetic impact projectiles (KIP) were used by law enforcement as a method of crowd control. We describe the injuries seen at a single Level 1 trauma center in Los Angeles over a two-day period of protests to add to the collective understanding of the public health ramifications of crowd-control weapons used in the setting of protests.

**Case Series:**

We reviewed the emergency department visits of 14 patients who presented to our facility due to injuries sustained from KIPs over a 48-hour period during civil protests after the death of George Floyd.

**Conclusion:**

Less lethal weapons can cause significant injuries and may not be appropriate for the purposes of crowd control, especially when used outside of established guidelines.

## INTRODUCTION

The United States saw a surge in the number of civil protests across the country following the death of George Floyd while in police custody. Law enforcement officers responded to these protests with crowd control measures, including in some instances the use of kinetic impact projectiles (KIP). As a result, protesters and bystanders alike suffered bodily injury ranging from superficial wounds to severe blunt and penetrating trauma resulting in injuries with potential for permanent disability. While protests are constitutionally protected and will always be part of our society, injuries associated with peaceful protests should not be. Thus, there is a need for further data regarding how crowd-control weapons (CCW) are used and the type and severity of injuries they cause, as well as appropriate recognition and management of these injuries by emergency physicians. We culled data from our institution to contribute to a collective understanding of the public health ramifications of CCW use in the setting of peaceful protests.

## CASE SERIES

We reviewed all emergency department (ED) visits at a Level I trauma center in Los Angeles, California, with proximity to the initial protests over the weekend of May 31-June 1, 2020, following the death of George Floyd on May 25. We searched for keywords suggesting traumatic injury either in the presenting complaint or final diagnosis. Upon review of those records captured by the criteria mentioned above, we further narrowed our search based on history and exam findings suggestive of traumatic injury inflicted by KIPs. Once cases were identified, we carefully reviewed each case, rating them for severity.

We defined minor injuries as those that were present on examination but did not require advanced professional medical care. These included contusions, abrasions, and sprains. Moderate injuries were defined as those requiring medical intervention, such as wounds and lacerations requiring suture repair and/or debridement, although the patients were safe for discharge from the ED with low potential for long-term disability. We defined severe injuries as those requiring operative management and/or hospitalization. A final category would include patients with injuries resulting in permanent disability or death.

We identified 14 patients who presented to our ED with injuries sustained from KIPs at protests in Los Angeles over the weekend of May 31–June 1, 2020. The average age of the cohort was 31 years (range 21–62). Five patients were women (36%), and nine were men. Two of the patients had injuries categorized as minor, while four patients had injuries considered to be moderate, and the remaining eight had injuries that were categorized as severe. Except for one patient with possible permanent vision loss who was subsequently lost to follow-up, none of our patients were permanently disabled and none of the injuries were lethal. Patient characteristics, injuries, and treatments are summarized in the table below.

The preponderance of our patients (78%) across all severity categories were treated for injuries sustained to the face and head. One patient in the severe category was treated for injury to the groin, while another in the severe category was treated for injury to the upper extremity. We were unable to confirm the exact type of projectile causing injury in any given case, although based on the pattern of injury, we suspect the majority to have been rubber bullets, with pellet rounds in three cases.

An unanticipated finding among three of our patients with soft tissue injuries was the presence of embedded, high-density (metal) foreign bodies in facial wounds noted on computed tomography imaging (Patients d, f. and j). These injuries are suspected to have been caused by “pellet rounds.” These CCWs are metal cartridge projectiles filled with pellets composed of lead, steel, or plastic/rubber that disperse on impact. A representative image is shown to the right.

## DISCUSSION

Crowd-control weapons including KIPs have traditionally been described as “less lethal” munitions, intended to incapacitate individuals posing immediate threat with pain or nonlethal injury when compared with live, or *lethal*, ammunition. There has been ongoing scrutiny and criticism over the use of KIPs, particularly in the setting of crowd control regarding their potential for disproportionate and indiscriminate use. More than 75 types of KIPs are available, among them both single-projectile weapons (rubber and plastic bullets), and scatter projectiles (bean bag, flash, or pellet rounds), all of which are sold to police, military, and private security operations around the world.^6^ Meanwhile, there is minimal discernible regulation when it comes to the manufacturing, marketing, and sales of these weapons.

Training for law enforcement on CCWs is unclear. A recent report found that while 40-millimeter rounds appeared to be responsible for most injuries sustained in Los Angeles during the George Floyd protests, Los Angeles Police Department (LAPD) officers were given only a one-time, two-hour training on the deployment of these munitions last in 2018. The report concludes that this level of training is woefully inadequate when considering the degree of skill and marksmanship required to use these weapons safely.^7^ While some guidelines on CCW use exist at the international level, federal and local guidelines are limited and there is evidence that even those are not well adhered to. There is no governing body or process of formal data collection regarding the injuries they have caused, and thus no procedures are in place for accountability on behalf of the companies that produce these weapons and the individuals who wield them.^5^ Most available data are limited to sparse case reports and case series in the medical literature,^6^ as well as coverage in the media and posts on social media accounts.

**CPC-EM Capsule**

What do we already know about this clinical entity?

*The use of kinetic impact projectiles (KIP) as “less lethal” weapons can result in serious bodily harm. The most significant injuries involve the head and neck.*

What makes this presentation of disease reportable?

*With the rise in number and frequency of protests worldwide, emergency physicians will likely see a rise in patients with injuries due to KIPs.*

What is the major learning point?

*The use of KIPs for crowd control may be inappropriate and unsafe from a public health perspective.*

How might this improve emergency medicine practice?

*Emergency physicians should become familiar with crowd-control weapons and the common injury patterns associated with their use.*

A 2017 review found that among 1984 KIP injuries reported in the literature over a period of 27 years from around the world, 30% were characterized as “minor,” while 70% were characterized as “severe,” and 3% resulted in death.^6^ These weapons have tremendous and unanticipated capacity for harm. Variables such as weapon shape and material, muzzle velocity, flight path, firing distance, and site of bodily impact influence not only injury severity, but target accuracy. Projectiles composed of dense materials such as metal will impart more force on impact. Others, designed with larger surface areas, have less predictable flight paths, especially when aimed from a distance. Inadequate understanding of these ballistic principles, including munition materials and their launching devices, by operators can lead to excessive harm to targeted individuals, as well as unintentional harm to bystanders. Much of the data previously reported suggest that injuries to the head and neck by KIPs tend to cause more harm than those to the limbs, leading to a recommendation by the United Nations Human Rights Council and some manufacturers that the face and neck should be avoided when aiming these weapons.^8^ The head, neck, spine, chest, groin, and kidneys are to be avoided according to LAPD policy guidelines as well. Our data reflect the importance of these guidelines.

## LIMITATIONS

There are several limitations in analyzing the data we collected. For one, our sample represents just a fraction of the injuries that were sustained across Los Angeles during the protests that weekend. Protests occurred in at least 49 cities in Los Angeles County, the nation’s largest county. Some individuals may have sought care at other nearby medical facilities, while many may never have sought care at all. According to a report that pooled social media posts from across the US, there were at least 12 KIP-inflicted head injuries in the Los Angeles area that weekend. and at least 115 people nationwide who were shot in the neck or head between May 26–July 27 at protests following George Floyd’s death in 2020.^9^ At least one patient in our series initially attributed her injuries to a fall, to conceal her participation in the protests from her parents at the bedside. We may have missed other, likely minor or moderate injuries in our review from patients who did not disclose accurate details regarding the mechanism of traumatic injury.

It is important to note that we were unable to ascertain the circumstances surrounding the injuries we treated, including whether KIPs were fired indiscriminately, at what range, aim, and with what degree of real and/or perceived threat from protesters claiming to be peaceful. To what degree these injuries correlate with significance of threat from protesters is unclear. Furthermore, we could neither verify what type of personnel (law enforcement, military officials and/or civilians) were wielding these weapons nor how many times such weapons were used that weekend. The release of reports from a formal investigation commissioned by the Los Angeles City Council is concomitant with the submission of this article and should shed more light on these areas of uncertainty.

## CONCLUSION

Based on the limited data obtained from patients seen at our facility treated for injuries sustained from KIPs during the George Floyd protests in Los Angeles, there is concern that these weapons can result in significant injuries and may not be appropriate for the purpose of crowd control. While some guidelines exist to use these weapons in a “less lethal” manner, there are limitations in how those guidelines are disseminated or followed by law enforcement. These data and their implications have been echoed in similar reports from other major cities in the US and worldwide. Since protests are a fixture of our society, these observations should be carefully considered by both the medical community and policymakers for the purpose of minimizing harm as law enforcement institutes crowd control measures.

## Figures and Tables

**Image:**
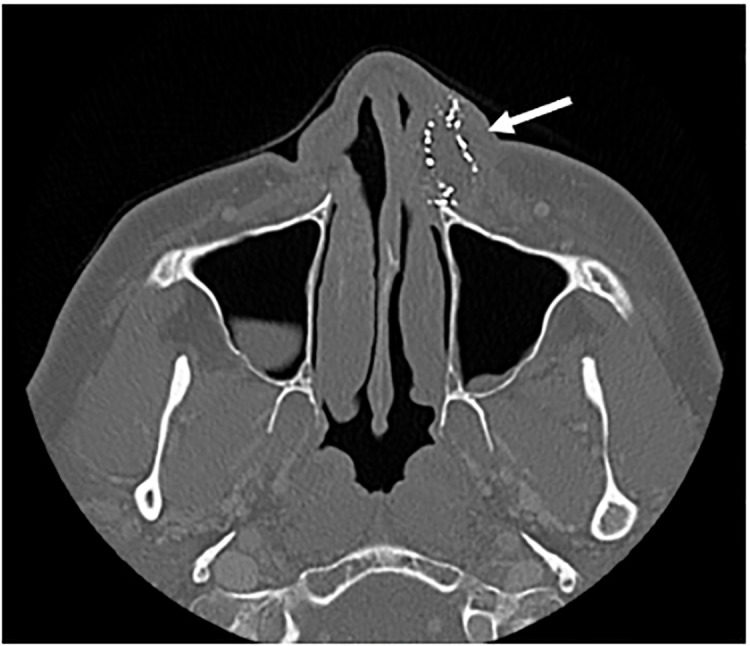
Computed tomography image of multiple embedded, high-density foreign bodies within left facial wound (arrow).

**Table:** Patient characteristics, injuries, and disposition from kinetic impact projectiles.

Patient	Age (yrs)	Gender	Purported Projectile	Injuries	Severity	Intervention	Hospitalized
a	24	Male	rubber bullet	Subarachnoid hemorrhage, subdural hematoma, parenchymal hemorrhagic contusions, facial laceration/contusion	severe	Intensive care unit stay, reversal of anticoagulationand suture repair	Yes
b	21	Male	rubber bullet	Open distal ulna fracture	severe	Surgical fixation	Yes
c	49	Male	rubber bullet	Testicular rupture	severe	Surgical repair	Yes
d	26	Female	pellet round	Maxillary fracture, facial laceration, soft tissue foreign body	severe	Complex wound repair	Yes
e	31	Female	rubber bullet	Nasal bone fractures, facial laceration	severe	Complex wound repair, nasal fracture reduction	No
f	27	Female	Pellet round	Complex facial lacerations, nasal fractures, soft tissue foreign body	severe	Complex wound repair	No
g	22	Female	rubber bullet/fall	Mandible fracture, lip laceration	severe	Surgical fixation, laceration repair	Yes
h	32	Female	rubber bullet	Orbital blowout fracture, hyphema, facial lacerations, corneal contusion	moderate	Visual loss (lost to follow up)	No
i	28	Male	rubber bullet	Facial bone fractures, facial laceration	moderate	Suture repair	No
j	62	Male	pellet round	Facial laceration, soft tissue foreign body, concussion	moderate	Suture repair	No
K	30	Male	rubber bullet	Scalp laceration, contusion of chest and neck	moderate	Suture repair	No
l	23	Male	rubber bullet	Face and arm lacerations, contusions	moderate	Suture repair	No
m	28	Male	rubber bullet	Dental fracture, facial contusion	minor	Dental referral	No
n	36	Male	rubber bullet	Contusions & abrasions to abdomen and lower extremity	minor	Wound care	No
